# Commentary: Inactivated rabies virus vectored MERS-Coronavirus vaccine induces protective immunity in mice, camels, and alpacas

**DOI:** 10.3389/fimmu.2025.1549481

**Published:** 2025-03-04

**Authors:** Shengnan Yang, Hanqi Li, Fuxiao Liu

**Affiliations:** ^1^ College of Veterinary Medicine, Qingdao Agricultural University, Qingdao, China; ^2^ Qingdao Center for Animal Disease Control & Prevention, Qingdao, China

**Keywords:** rabies virus, MERS-CoV, inactivated RABV-vectored vaccine, antibody, immune response

## Introduction

A research article, named *Inactivated Rabies Virus Vectored MERS-Coronavirus Vaccine Induces Protective Immunity in Mice, Camels, and Alpacas* (doi: 10.3389/fimmu.2022.823949), was recently published in *Frontiers in Immunology* (Section: Vaccines and Molecular Therapeutics) (1). In this study, Chi et al. constructed a chimeric rabies virus (RABV) that expressed a genetically modified S1 gene from the Middle East respiratory syndrome coronavirus (MERS-CoV), and then evaluated its potential of virus-vectored vaccine after inactivation in different animals. This study demonstrated that the inactivated S1-expressing RABV was a promising vaccine candidate against MERS-CoV for camelids. Here, we would like to express our scientific opinions on this study.

## Middle East respiratory syndrome

MERS is a severe infectious disease caused by MERS-CoV, initially identified in Saudi Arabia in 2012 (2). Typical signs clinically include fever, cough and shortness of breath in humans. MERS-CoV is a zoonotic virus, which has been identified in dromedary camels in several Member States in the Middle East, Africa and South Asia (3). There are a number of candidate vaccines that have been reported against MERS-CoV, including nucleic acid vaccine (4), subunit vaccine (5), nanoparticle vaccine (6), virus-vectored vaccine (7), and even live-attenuated vaccine (8). Most of the candidate vaccines have been designed using the MERS-CoV S protein, especially the S1 subunit.

## Development of RABV-vectored vaccines

RABV virion is a bullet-shaped particle, containing a single-stranded, negative-sense RNA genome, coding for five proteins in order: nucleoprotein (N), phosphoprotein (P), matrix protein (M), glycoprotein (G), and RNA-dependent RNA polymerase (L). The RABV can be genetically modified using reverse genetics, whereby a foreign sequence can be inserted into the RABV genome for rescuing a replication-competent chimeric virus (9). This genetically modified RABV, if demonstrated to be able of expressing a foreign antigen that induces immune responses *in vivo*, would play a potential role in the development of RABV-vectored vaccines, such as live-attenuated vaccines (10, 11), inactivated vaccines (12, 13) and replication-deficient vaccines (14, 15).

## Characteristics of chimeric RABV for developing inactivated vaccine

**Figure 1:**
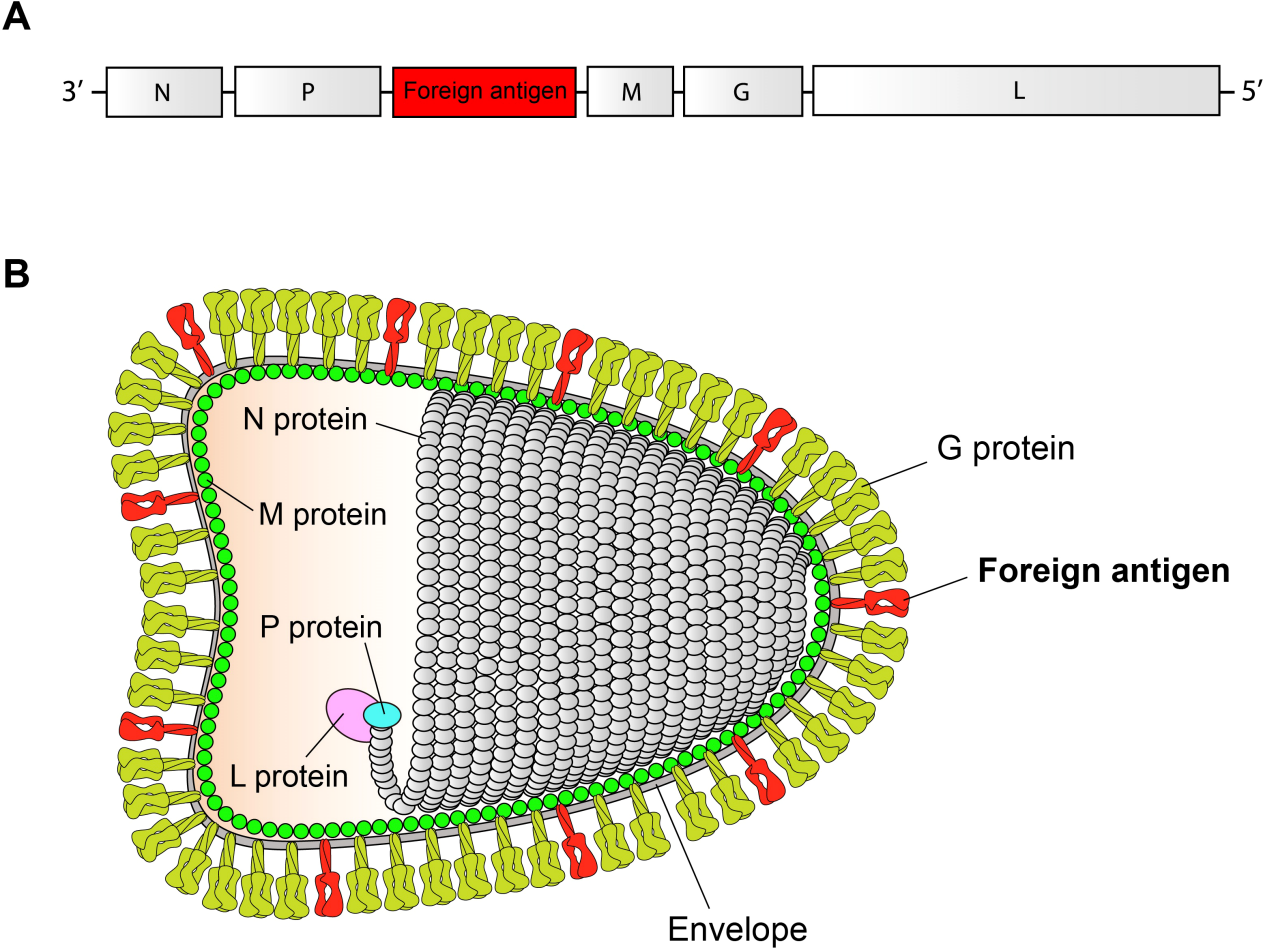
Schematic representations of chimeric RABV genome and virion. Chimeric RABV genome **(A)**. The chimeric genome contains a single transcription unit of foreign antigen. Chimeric RABV virion **(B)**. The foreign antigen, as a membrane-spanning protein, is embedded into the RABV envelope.

The inactivated RABV-vectored vaccine (IRVV) is a killed version of antigen-expressing RABV, rescued from its cDNA clone using reverse genetics. The development of an IRVV involves the construction of a chimeric RABV cDNA clone, which contains a foreign gene for virus recovery. More importantly, it must be ensured that the target antigen can be incorporated into the envelope of RABV virion (16). The foreign sequence-containing genome and the foreign antigen-containing virion are schematically shown in **Figures 1A, B**, respectively. Some RABV-expressed antigens will be processed, transported to the cell surface, and finally, as membrane-spanning proteins, embedded into the cellular envelope. Along with budding of RABV virion, the foreign antigen can be incorporated into the viral envelope (13, 17).

## Development of anti-MERS-CoV IRVV

In the article published by Chi et al., the sequence of MERS-CoV S1 subunit was infused with the transmembrane domain (TM) of human CD4 and the cytoplasmic domain (CD) of RABV G protein. The fusion fragment, MERS_S1_-TM-CD, as a single transcription unit was inserted into the RABV (SRV9 strain) cDNA clone for rescuing a chimeric RABV, rSRV9-MERS_S1_. The transmission electron microscopy exhibited that the viable virus was successfully rescued using reverse genetics. The indirect immunofluorescence assay confirmed that the S1 subunit was expressed and transported to the cell surface. Subsequently, the rSRV9-MERS_S1_ stock was harvested, inactivated by the β-propiolactone, and then purified by the ultracentrifugation on a discontinuous sucrose gradient.

Further, Chi et al. carried out *in vivo* tests using three different animals: mice, camels, and alpacas. The test in mice revealed that the inactivated rSRV9-MERS_S1_ induced not only the robust, specific responses of MERS-CoV antibodies, but also the CD8^+^ T cell-specific responses. Moreover, the immunization alleviated virus replication and quickened virus clearance in MERS-CoV-infected mice. In addition, humoral immunities against MERS-CoV and RABV were identified in rSRV9-MERS_S1_-vaccinated camels and alpacas. The immune sera had a wide range of cross-neutralizing antibody responses against three MERS-CoV clades in camels and alpacas. Furthermore, MERS-CoV-specific variable domains of heavy-chain-only antibody were isolated from vaccinated alpacas and demonstrated to have robust therapeutic, prophylactic efficacies in a genetically modified mouse model.

## Discussion

Compared with the live-attenuated vaccine, the IRVV shows a good safety profile *in vivo*, as evidenced by neither virus mutation nor virulence reversion occurring in IRVV-vaccinated animals. A chimeric RABV, albeit chemically inactivated, can even completely retain its immunogenicity, eliciting not only the anti-RABV immune response, but also more significantly, high-level antibodies against a target pathogen. In the study conducted by Chi et al., the rSRV9-MERS_S1_-inoculated mice, camels and alpacas were independently demonstrated to be able of secreting MERS-CoV-specific antibodies, implying its ability to inhibit the MERS-CoV infection in animals.

It has been widely demonstrated that inactivated chimeric RABVs have promising potentials in developing IRVVs (1, 12, 13, 17–23), whereas there are still a few disadvantages to them. For example, IRVVs may be less efficient than live-attenuated RABV-vectored vaccines in immunogenicity. The latter can elicit so potent immune response that a single dose is sufficient for the vaccination of animals, whereas the former are generally involved in the prime–boost vaccination (24, 25). In order to obtain MERS-CoV-specific antibodies at a high level, mice, camels and alpacas were separately subjected to the prime–boost vaccination in the study conducted by Chi et al. The test of MERS-CoV challenge showed that the rSRV9-MERS_S1_-based vaccination reduced MERS-CoV replication and accelerated its clearance in the lungs of genetically modified mice. Unfortunately, neither camels nor alpacas were subjected to the test of MERS-CoV challenge.

MERS-CoV has been still regarded as an emerging virus. Although various anti-MERS-CoV candidate vaccines have been recently reported, none of them has been commercially available as yet. In the field of virus-vectored vaccines, Chi et al. carried out their study that was valuable for guiding the development of IRVV. In conclusion, the inactivated rSRV9-MERS_S1_ was safe for the vaccine-inoculated animals, and induced potent immune responses *in vivo*. Therefore, this study will pave the way for the construction of virus-vectored vaccines against MERS-CoV in future.

## References

[1] Chi H Wang Y Li E Wang X Wang H Jin H. Inactivated rabies virus vectored MERS-coronavirus vaccine induces protective immunity in mice, camels, and alpacas. Front Immunol. (2022) 13:823949. doi: 10.3389/fimmu.2022.823949 35173733 PMC8842186

[2] Bermingham A Chand MA Brown CS Aarons E Tong C Langrish C. Severe respiratory illness caused by a novel coronavirus, in a patient transferred to the United Kingdom from the Middle East, September 2012. Euro Surveill. (2012) 17:20290. doi: 10.2807/ese.17.40.20290-en 23078800

[3] Peiris M Perlman S. Unresolved questions in the zoonotic transmission of MERS. Curr Opin Virol. (2022) 52:258–64. doi: 10.1016/j.coviro.2021.12.013 PMC8734234 34999369

[4] Almansour I Jermy BR. Nucleic acid vaccine candidates encapsulated with mesoporous silica nanoparticles against MERS-CoV. Hum Vaccin Immunother. (2024) 20:2346390. doi: 10.1080/21645515.2024.2346390 38691025 PMC11067998

[5] Zhang N Tang J Lu L Jiang S Du L. Receptor-binding domain-based subunit vaccines against MERS-CoV. Virus Res. (2015) 202:151–9. doi: 10.1016/j.virusres.2014.11.013 PMC4439384 25445336

[6] Hashemzadeh A Avan A Ferns GA Khazaei M. Vaccines based on virus-like nano-particles for use against Middle East Respiratory Syndrome (MERS) coronavirus. Vaccine. (2020) 38:5742–6. doi: 10.1016/j.vaccine.2020.07.003 PMC7837099 32684497

[7] Malczyk AH Kupke A Prüfer S Scheuplein VA Hutzler S Kreuz D. A highly immunogenic and protective middle east respiratory syndrome coronavirus vaccine based on a recombinant measles virus vaccine platform. J Virol. (2015) 89:11654–67. doi: 10.1128/JVI.01815-15 PMC4645655 26355094

[8] Gutiérrez-Álvarez J Honrubia JM Fernández-Delgado R Wang L Castaño-Rodríguez C Zúñiga S. Genetically engineered live-attenuated middle east respiratory syndrome coronavirus viruses confer full protection against lethal infection. mBio. (2021) 12:e00103–21. doi: 10.1128/mBio.00103-21 PMC8092200 33653888

[9] Osakada F Callaway EM. Design and generation of recombinant rabies virus vectors. Nat Protoc. (2013) 8:1583–601. doi: 10.1038/nprot.2013.094 PMC4028848 23887178

[10] Xu T Liu L Shi C Liu W Wang M Tian L. A recombinant rabies virus expressing Echinococcus granulosus EG95 induces protective immunity in mice. Transbound Emerg Dis. (2022) 69:e254–e66. doi: 10.1111/tbed.14292 34403194

[11] Kurup D Wirblich C Feldmann H Marzi A Schnell MJ. Rhabdovirus-based vaccine platforms against henipaviruses. J Virol. (2015) 89:144–54. doi: 10.1128/JVI.02308-14 PMC4301098 25320306

[12] Jiao C Liu D Jin H Huang P Zhang H Li Y. Immunogenicity evaluation of a bivalent vaccine based on a recombinant rabies virus expressing gB protein of FHV-1 in mice and cats. Vet J. (2024) 304:106096. doi: 10.1016/j.tvjl.2024.106096 38503385

[13] Abreu-Mota T Hagen KR Cooper K Jahrling PB Tan G Wirblich C. Non-neutralizing antibodies elicited by recombinant Lassa-Rabies vaccine are critical for protection against Lassa fever. Nat Commun. (2018) 9:4223. doi: 10.1038/s41467-018-06741-w 30310067 PMC6181965

[14] Takayama-Ito M Lim CK Yamaguchi Y Posadas-Herrera G Kato H Iizuka I. Replication-incompetent rabies virus vector harboring glycoprotein gene of lymphocytic choriomeningitis virus (LCMV) protects mice from LCMV challenge. PloS Negl Trop Dis. (2018) 12:e0006398. doi: 10.1371/journal.pntd.0006398 29659579 PMC5901774

[15] Gomme EA Faul EJ Flomenberg P McGettigan JP Schnell MJ. Characterization of a single-cycle rabies virus-based vaccine vector. J Virol. (2010) 84:2820–31. doi: 10.1128/JVI.01870-09 PMC2826042 20053743

[16] Scher G Schnell MJ. Rhabdoviruses as vectors for vaccines and therapeutics. Curr Opin Virol. (2020) 44:169–82. doi: 10.1016/j.coviro.2020.09.003 PMC8331071 33130500

[17] Shuai L Wang X Wen Z Ge J Wang J Zhao D. Genetically modified rabies virus-vectored Ebola virus disease vaccines are safe and induce efficacious immune responses in mice and dogs. Antiviral Res. (2017) 146:36–44. doi: 10.1016/j.antiviral.2017.08.011 28822816

[18] Smith ME Koser M Xiao S Siler C McGettigan JP Calkins C. Rabies virus glycoprotein as a carrier for anthrax protective antigen. Virology. (2006) 353:344–56. doi: 10.1016/j.virol.2006.05.010 PMC1576297 16820183

[19] Rios S Bhattachan B Vavilikolanu K Kitsou C Pal U Schnell MJ. The Development of a Rabies Virus-Vectored Vaccine against *Borrelia burgdorferi*, Targeting BBI39. Vaccines (Basel). (2024) 12:78. doi: 10.3390/vaccines12010078 38250891 PMC10820992

[20] Kurup D Fisher CR Scher G Yankowski C Testa A Keshwara R. Tetravalent rabies-vectored filovirus and lassa fever vaccine induces long-term immunity in nonhuman primates. J Infect Dis. (2021) 224:995–1004. doi: 10.1093/infdis/jiab014 33421072 PMC8448432

[21] Huang J Wang W Li H Bai Y Song Y Jiao C. Three in one: An effective and universal vaccine expressing heterologous tandem RBD trimer by rabies virus vector protects mice against SARS-CoV-2. Antiviral Res. (2024) 227:105905. doi: 10.1016/j.antiviral.2024.105905 38740191

[22] Zhang H Jin H Yan F Song Y Dai J Jiao C. An inactivated recombinant rabies virus chimerically expressed RBD induces humoral and cellular immunity against SARS-CoV-2 and RABV. Virol Sin. (2023) 38:244–56. doi: 10.1016/j.virs.2022.12.006 PMC9797420 36587795

[23] Zhang S Hao M Feng N Jin H Yan F Chi H. Genetically modified rabies virus vector-based rift valley fever virus vaccine is safe and induces efficacious immune responses in mice. Viruses. (2019) 11:919. doi: 10.3390/v11100919 31597372 PMC6832564

[24] Keshwara R Shiels T Postnikova E Kurup D Wirblich C Johnson RF. Rabies-based vaccine induces potent immune responses against Nipah virus. NPJ Vaccines. (2019) 4:15. doi: 10.1038/s41541-019-0109-5 31016033 PMC6465360

[25] Keshwara R Hagen KR Abreu-Mota T Papaneri AB Liu D Wirblich C. A Recombinant Rabies Virus Expressing the Marburg Virus Glycoprotein Is Dependent upon Antibody-Mediated Cellular Cytotoxicity for Protection against Marburg Virus Disease in a Murine Model. J Virol. (2019) 93:e01865–18. doi: 10.1128/JVI.01865-18 PMC6401435 30567978

